# A prognostic nomogram for overall survival after neoadjuvant radiotherapy or chemoradiotherapy in thoracic esophageal squamous cell carcinoma: a retrospective analysis

**DOI:** 10.18632/oncotarget.17062

**Published:** 2017-04-12

**Authors:** Wei Deng, Qifeng Wang, Zefen Xiao, Lijun Tan, Zhao Yang, Zongmei Zhou, Hongxing Zhang, Dongfu Chen, Qinfu Feng, Jun Liang, Yexiong Li, Jie He, Shugeng Gao, Kelin Sun, Guiyu Cheng, Xiangyang Liu, Dekang Fang, Qi Xue, Yousheng Mao, Dali Wang, Jian Li

**Affiliations:** ^1^ Department of Radiation Oncology, National Cancer Center/Cancer Hospital, Chinese Academy of Medical Sciences and Peking Union Medical College, Beijing, China; ^2^ Department of Radiation Oncology, Sichuan Cancer Hospital, Chengdu, China; ^3^ Department of Oncology, First Hospital of Harbin Medical College, Harbin, China; ^4^ Department of Cancer Epidemiology, National Cancer Center/Cancer Hospital, Chinese Academy of Medical Sciences and Peking Union Medical College, Beijing, China; ^5^ Department of Thoracic Surgery, National Cancer Center/Cancer Hospital, Chinese Academy of Medical Sciences and Peking Union Medical College, Beijing, China

**Keywords:** esophageal carcinoma, nomogram, neoadjuvant therapy, overall survival, recursive partitioning analysis

## Abstract

**Background:**

Currently, the AJCC staging system or pathological complete response (pCR) are considered not sufficiently accurate to evaluate the survival of patients with esophageal squamous cell carcinoma after neoadjuvant radiotherapy or chemoradiotherapy. This study aimed to establish a nomogram and a recursive partitioning analysis (RPA) model to estimate prognosis and to provide advice for subsequent treatments.

**Methods:**

We analyzed retrospectively 407 patients that were diagnosed with thoracic esophageal squamous cell carcinoma (TESCC) and received neoadjuvant radiotherapy or chemoradiotherapy. Hazard ratios and 95% confidence intervals of categorical clinicopathological characteristics with overall survival (OS) were calculated using the Cox proportional hazard model. The nomogram and RPA model were then established and total scores according to each variable were calculated and stratified to predict OS.

**Results:**

Patients were followed-up over a median 49.9 months. AJCC did not perform well in distinguishing OS among each stage except for IIB and IIIA. Patients were divided into 4 groups according to the total scores based on nomogram (low risk: ≤180; intermediate risk: 180-270; high risk: 270-340; very high risk: >340). The 5-year OS was 57.3%, 40.7%, 18.3%, 6.1% respectively (p<0.05). RPA model also divide the patients into 4 groups, though group2 and group3 were not statistically significant (p=0.574).

**Conclusion:**

The nomogram is a good evaluation model for estimating the prognosis of patients with TESCC after neoadjuvant radiotherapy or chemoradiotherapy compared with the AJCC and RPA. The results of this study also suggested that the high-risk subgroups need further treatments.

## INTRODUCTION

Esophageal cancer is the 3^rd^ most common cancer in China, with an estimated 477,900 new cases in 2015, and is the 4^th^ most common cause of death worldwide, with an estimated 375,000 deaths in 2015 [1] Nearly 50 % of esophageal cancer worldwide occurs in China [2], and the majority of esophageal cancer in China is esophageal squamous cell carcinoma, which accounts for over 90 % of new cases [3].

Previous studies have demonstrated that the addition of neoadjuvant chemoradiotherapy (NCRT) to surgery results in an increase in the R0 resection rate and survival, with acceptable perioperative morbidities [4–6]. However, the 3-year overall survival of these patients varies from 17 % to 63.5 % because of the different and mutual effects of the treatments [7, 8]. Therefore, it is essential to restage these patients accurately after completion of NCRT and to provide more information for subsequent treatment.

The American Joint Committee on Cancer (AJCC) staging system for esophageal cancer is used widely for cancer staging and is based on the retrospective analysis of pathological data from surgical resection. This staging system is controversial because it includes little consideration of the effects of neoadjuvant therapy [9, 10]. Arguments have focused on the diverse treatment responses of primary tumors and lymph nodes, resulting in different regression grade, and that differ widely from the pretreatment status, consequently it would be inappropriate to use the AJCC staging system for patients who received NCRT. Another frequently used criterion is the pathological complete response (pCR). Patients with a pCR tend to experience better prognoses [11–15]. However, the non-pCR patients (accounting for 70 %) exist as diverse subsets. Wang et al. [16] proposed that the presence of residual lymph nodes is an independent prognostic factor in pT0 esophageal cancer after preoperative radiotherapy. Similarly, survival in the pT1-2N0 stage and pT3-4N+ stage is different; therefore, multiple factors must exist that separate different prognostic subsets precisely.

A nomogram is an advanced and widely applied model that estimates the survival of an individual patient by incorporating multiple variables and their interdependent relationships. Nomograms have been used to stratify and predict prognosis precisely in several cancers [17–19]. Recursive partitioning analysis (RPA) is another model for risk stratification, which is easy to use and has been introduced to many cancer areas [20, 21]. The only weak point is that the RPA model lacks the ability to precisely associate risk with survival. To the best of our best knowledge, these two methods have not been discussed at the same time in terms of neoadjuvant therapy of ESCC patients.

Therefore, this study aimed to establish a model to estimate prognosis using a nomogram, to compare the predicative efficacy and stratification ability of the nomogram with other models, and to provide advice for subsequent treatment.

## RESULTS

### Baseline characteristics

From January 1980 to December 2014, 407 eligible patients were enrolled. The median follow up time was 26.0 months (49.9 months for censor cases) with 46 % of the follow up time exceeding 60 months and 62.2 % OS events being reached. Table 1 shows the demographic characteristics and clinical characteristics of the 407 patients. There were more men than women (ratio, 4.2:1). The median age was 56 years (range, 27–78 years). The middle section was the most common site for the primary tumor and 79.4 % of patients had a tumor longer than 5 cm based on endoscopy. More patients received conventional radiotherapy (ratio, 3.0:1). Radical resection was applied in 83.8 % patients. Among all the patients, 163 (40.0 %) achieved complete response of the primary tumor; 146 (35.9 %) achieved a partial response; and 98 (24.1 %) showed a minimal response. A total of 5544 nodes were dissected (median number of 12 per patient), and the proportion of pathological lymph nodes was 30.7 %. Postoperative anastomotic leakage occurred in 29 patients (7.1 %). The mortality rate within 30 days after surgery was 3.9 % (n=16). The 5-year OS rate and disease free survival (DFS) were 36.7 % and 36.1 %, respectively. According to different decades, patients enrolled in 1980–1989, 1990–1999, and 2000–2014 had a 5-year OS of 33.7 %, 32.6 %, and 44.1 %; and a median overall survival of 26.4 months, 27.4 months, and 42.5 months, respectively. Patients enrolled in 2000–2014 had the best survival compared with the other two groups (p < 0.05).

**Table 1:** Baseline Characteristics of the Patients

Characteristic	Score ≤ 180(n = 129)		Score 180–270(n = 147)		Score 270–340(n = 65)		Score > 340(n = 66)		All patients(n = 407)		*P*
	No.	%	No.	%	No.	%	No.	%	No.	%	
Sex											<0.001
Male	86	66.7	123	83.7	59	90.8	61	92.4	329	80.8	
Female	43	33.3	24	16.3	6	9.2	5	7.6	78	19.2	
Age (years)											<0.001
< 55	68	52.7	58	39.5	35	53.8	16	24.2	177	43.5	
≥ 55	61	47.3	89	60.5	30	46.2	50	75.8	230	56.5	
Median	54		56		53		58		56		
Tumor length											<0.001
< 5 cm	48	37.2	25	17.0	8	12.3	3	4.5	84	20.6	
≥ 5 cm	81	62.8	122	83.0	57	87.7	63	95.5	323	79.4	
Tumor Section											0.694
Upper	24	18.6	27	18.4	9	13.8	12	18.2	72	17.7	
Middle	89	69.0	100	68.0	46	70.8	50	75.8	285	70.0	
Lower	16	12.4	20	13.6	10	15.4	4	6.1	50	12.3	
Treatment modality											
Radiotherapy	91	70.5	124	84.4	53	81.5	61	92.4	329	80.8	0.001
Chemoradiotherapy	38	29.5	23	15.6	12	18.5	5	7.6	78	19.2	
Radiation modality											<0.001
Conventional RT	79	61.2	117	79.6	52	80.0	58	87.9	306	75.2	
3DCRT/IMRT	50	38.8	30	20.4	13	20.0	8	12.1	101	24.8	
Total dose(Gy)											-
Median(except SIB)	40		40		40		40				
Resection margin											<0.001
Radical	128	99.2	132	89.8	49	75.4	32	48.5	341	83.8	
Palliative	1	0.8	15	10.2	16	24.6	34	51.5	66	16.2	
Proximal margin length											<0.001
< 4 cm	28	21.7	70	47.6	31	47.7	48	72.7	177	43.5	
≥ 4 cm	101	78.3	77	52.4	34	52.3	18	27.3	230	56.5	
Treatment response											<0.001
Complete	83	64.3	59	40.1	16	24.6	5	7.6	163	40.0	
Partial	45	40.1	59	40.1	24	36.9	18	27.3	146	35.9	
Minimal	1	24.6	29	19.7	25	27.3	43	65.2	98	24.1	
Lymph node status											<0.001
Negative	125	96.9	116	78.9	23	35.4	18	27.3	282	69.3	
Positive	4	3.1	31	21.1	42	64.6	48	72.7	125	30.7	
Anastomotic leakage											<0.001
No	129	100	137	93.2	61	93.8	51	77.3	378	92.9	
Yes	0	0	10	6.8	4	6.2	15	22.7	29	7.1	

(3DCRT, 3-dimensional conformal radiotherapy; IMRT, intensity modulated radiotherapy; SIB, simultaneously integrated boost)

### Prognostic nomogram

**Figure 1:**
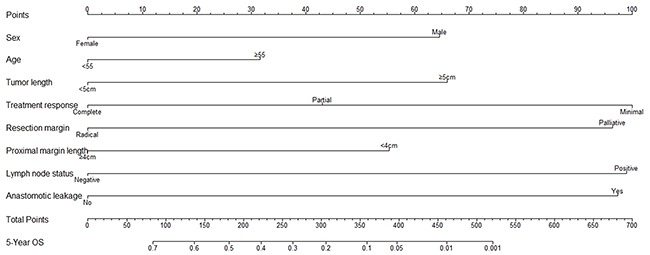
Prognostic nomogram for overall survival of esophageal carcinoma patients after neoadjuvant radiotherapy or chemoradiotherapy The scores for each variable attributed to an individual patient are located on the corresponding axis, and a line is drawn upwards to determine the number of scores received for each variable. The sum of these numbers is located on the total points axis, and a line is drawn downward to the survival axis to determine the likelihood of 5-year survival.

Nomogram construction was based on the Cox proportional hazard model, showing that sex, tumor length (<5 cm and ≥ 5 cm), treatment response, resection margin, proximal margin length (< 4 cm and ≥ 4 cm), lymph node status, and anastomotic leakage predicted OS independently. However, age was also added for prognostic model building because of its clinical relevance (Table 2). Points for these independent factors were assigned according to their coefficients. The probability of 5-year OS was determined by the total number of points, which was the sum of all points (Figure 1). The patients were then divided into four groups according to the total scores (total scores: low risk group: ≤ 180; intermediate risk group: 180–270; high risk group: 270–340; very high risk group: > 340). The 5-year OS rates were 57.3 %, 40.7 %, 18.3 %, and 6.1% (low risk *vs*. intermediate risk, p = 0.025; intermediate risk *vs*. high risk, p=0.01; high risk *vs*. very high risk, p=0.000; Figure 3D), respectively. When applying this model to DFS, it also showed significant differences between groups. The 5-year DFS rates were 57.4 %, 40.8 %, 18.3 %, and 6.0 % (low risk *vs*. intermediate risk, p=0.024; intermediate risk *vs*. high risk, p=0.002; high risk *vs*. very high risk, p=0.000; Figure 3E), respectively.

**Table 2:** Multivariate Analysis of Overall Survival in 407 patients

Variable	*HR*	*95 % CI*	*P-Value*
Sex	1.55	1.10–2.18	0.0120
Age	1.24	0.95–1.61	0.1078
Tumor length	1.56	1.10–2.23	0.0137
Treatment response			
Partial *vs*. Complete	1.34	0.98–1.83	0.0670
Minimal *vs*. Complete	1.97	1.40–2.75	<0.0001
Resection margin	1.92	1.37–2.69	0.0001
Proximal margin length	1.46	1.12–1.89	0.0049
Lymph node status	1.96	1.48–2.58	<0.0001
Anastomotic leakage	1.93	1.20–3.12	0.0067

**Figure 2:**
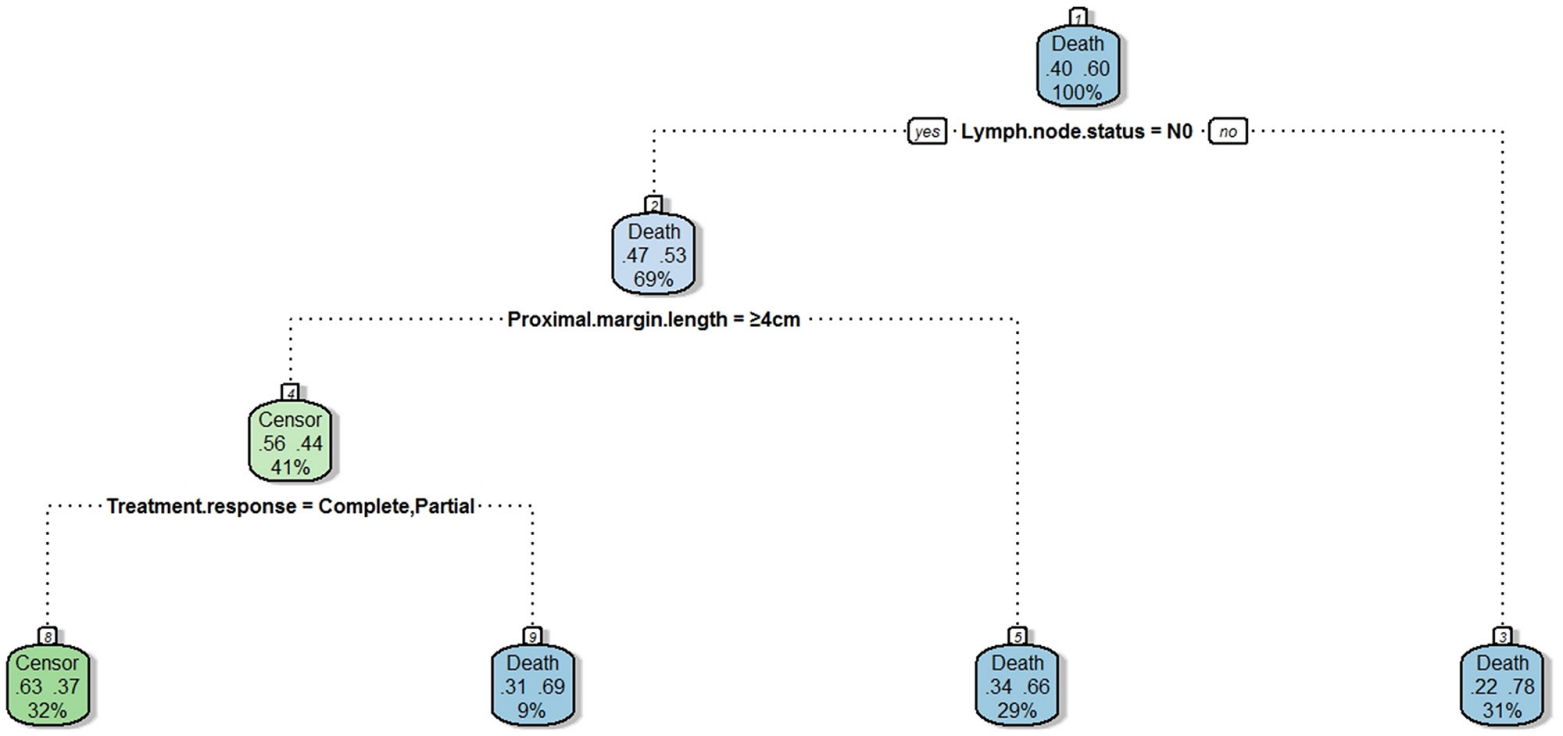
Recursive partitioning analysis for overall survival of 407 esophageal carcinoma patients The patients who had pathological positive lymph nodes (group 4) had the worst survival, which accounted for 31 %. Patients who had negative lymph nodes and proximal margin length < 4 cm showed better survival (group 3), which accounted for 29 %. Patients who had negative lymph nodes, proximal margin length ≥ 4 cm and minimal treatment response had the second best survival (group 2), which accounted for 9 %. The remaining patients who had negative lymph nodes, proximal margin length ≥ 4 cm and complete or partial treatment response had the best survival (group 1), which accounted for 32 %.)

**Figure 3:**
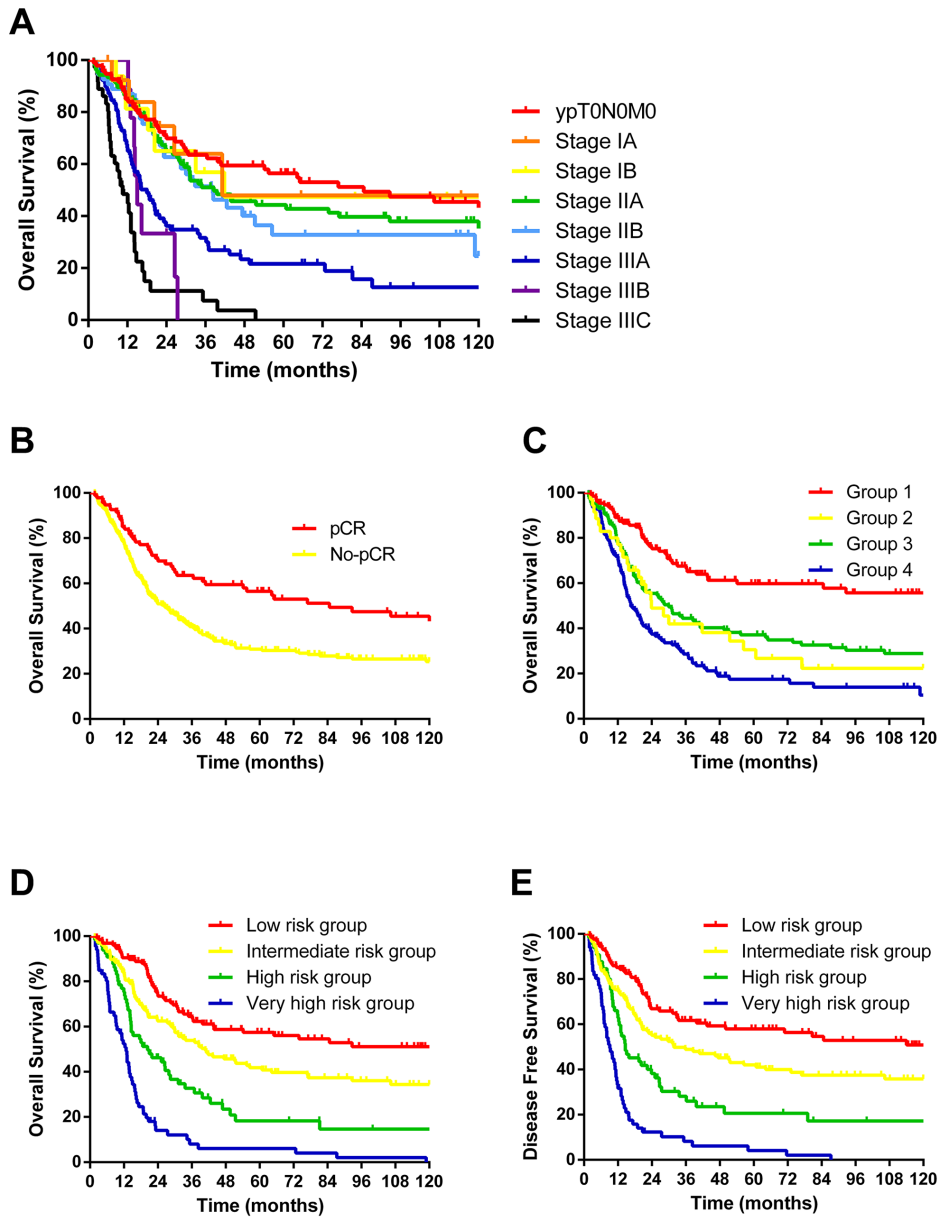
**(A)** 5-year OS of 407 patients according to 7^th^ AJCC staging system. Each stage was not distinguished from the other except for IIb and IIIA; **(B)** 10-year OS of 407 patients according to pCR. The two groups differed significantly but overlooked potential subgroups; **(C)** 10-year OS of 407 patients according to RPA. Groups 2 and 3 did not differ significantly; **(D)** 10-year OS of 407 patients according to the nomogram; **(E)** 10-year DFS of 407 patients according to the nomogram. Both D and E showed an excellent effect of survival stratification.

**Figure 4:**
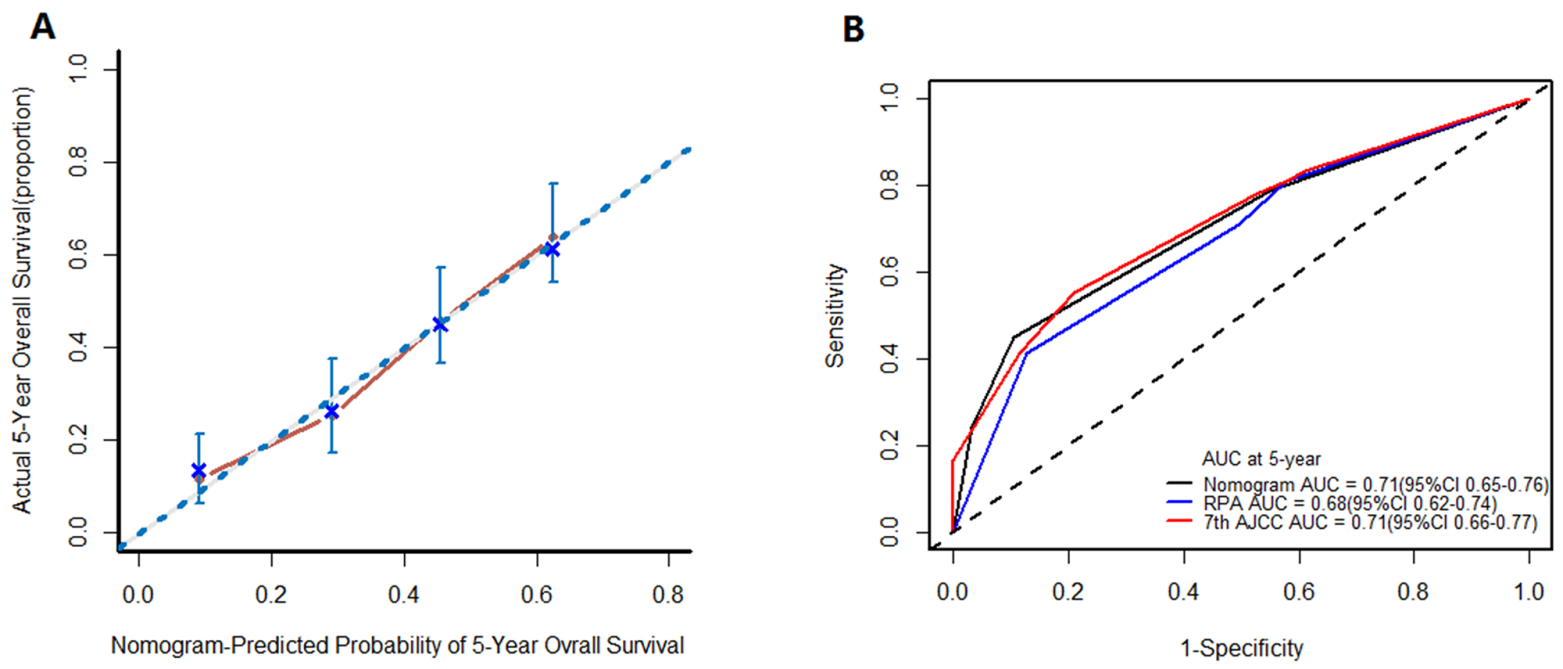
**(A)** Calibration plot for the nomogram. The 45° line represents the ideal predictions, and this plot shows a high degree of similarity between the actual and the estimated survival rate; **(B)** ROC curves for nomogram, AJCC, and RPA. The ROC curves presented higher sensitivity and specificity at 1 and 3 years, but were similar at 5 years to that of the AJCC.

A calibration curve was constructed that compared the nomogram predicted probabilities of OS with actual survival at year 5 (Figure 4A). We observed a high degree of similarity between the observed and the estimated rate (the 45° line represents ideal predictions).

### RPA modeling

Analysis of OS using RPA and multivariate factors as variables showed that patients could be stratified to four prognostic groups according to lymph node status, proximal margin length, and treatment response (the only factors that were available to stratify patient survival in this model). Those who had pathological lymph node metastasis were identified as group 4; patients with tumors with a proximal margin length < 4 cm were identified as group 3; patients achieving a minimal pathological response were identified as group 2; patients achieving a complete or partial response were identified as group 1 (Figure 2). The RPA model also showed good identification efficacy; however, group 2 and 3 were not statistically significant for OS (p=0.574; Figure 3C).

### AJCC cancer staging system and pCR

According to the 7^th^ AJCC Cancer staging system, 95 patients had a pCR and thus were defined as ypT0N0M0; 14 had stage IA disease, 16 had stage IB, 105 had stage IIA, 54 had stage IIB, 78 had stage IIIA, nine had stage IIIB, and 36 had stage IIIC. The AJCC Cancer staging system did not perform well in distinguishing the OS among each stage, except for stages IIB and IIIA (p = 0.005, Figure 3A). Patients that achieved a pCR had a 5-year OS of 56.5 % and a median survival time of 84.7 months, compared with 30.8 % and 26.4 months for non-pCR patients (p = 0.001, Figure 3B). The 5-year OS for patients with a complete, partial, and minimal response were 50.9 %, 35.5 %, and 16.3%; the median survival times were 64.2 months, 31.3 months, and 15.9 months, respectively (p = 0.000).

### Comparison of the predicative efficacy of risk for OS

The prognostic nomogram showed better predicative efficacy and risk stratification ability compared with the RPA and AJCC methods. The c-index of the nomogram was 0.67, which was slightly higher than that of the AJCC staging system (0.64) and RPA (0.62). The ROC curves also showed higher sensitivity and specificity at 1 and 3 years, as measured by area under the curve (AUC). However, the nomogram presented similar results for OS at 5 years to that of AJCC (0.71, 95 %CI: 0.65–0.76 *vs*. 0.71, 95 %CI: 0.66–0.77; Figure 4B), which might indicate that this nomogram has a better performance for survival prediction and risk stratification than the other two methods, at least in the first 5 years. This analysis also suggested the instability of the AJCC staging system.

## DISCUSSION

Neoadjuvant chemoradiotherapy is recommended for resectable esophageal carcinoma (stages T1bN+, T2-4aN0-N+, and M0) in the NCCN guidelines, mainly based on the evidence of relevant meta-analysis and the CROSS study [4–6]. However, even patients assessed as being at the same stage before treatment could experience different prognosis because of diverse sensitivities to chemoradiotherapy. Although NCRT is an established approach for resectable TESCC, there seems to be no consensus regarding prognosis stratification and prediction after NCRT.

Use of the AJCC staging system after NCRT was not recommended in several studies [9, 10], because it attaches much importance to the TNM stage, and ignores age, tumor length, surgical information, and other prognostic variables, and thus did not show good correspondence with survival in our research data. Rizk et al. [9] enrolled 276 esophageal cancer patients to receive NCRT; except for 52 that had a pCR, the remaining non-pCR patients were categorized from stage I to IV according to the AJCC staging system. It turned out that AJCC did not discriminate well between patients who were staged pCR and IIA (p= 0.52), patients who were staged IIB and III (p= 0.87), and patients who were staged IVA and IVB (p= 0.30). The authors concluded that the AJCC staging system does not adequately stratify prognostic groups.

Many studies have shown that pCR is a good indicator of a better prognosis. However, the crude classifying of patients into pCR and residual disease is overly simplistic. As proposed by several studies, although the primary tumor reached pCR in some cases, the presence of residual lymph nodes was an independent prognostic factor that increases the risk of recurrence after neoadjuvant therapy [16, 22, 23]. In Wang’ study [16], N0 diseases had a significantly improved 5-year OS and DFS compared with N+ diseases. Another study claimed that even though they reached pathological ypT0, patients with residual lymph nodes had a poor prognosis and behaved similarly to pathological stage II/III disease [22]. Other clinicopathological parameters, such as sex, age, and tumor grade [24, 25] have been recognized as important predictors of survival. Thus, pCR and non-pCR should not simply be adopted without further stratification.

Nomograms have been constructed in many malignancies, some of which have been found to be more reliable than the traditional staging systems (UICC or AJCC). Our study established a prognostic nomogram that could stratify patients that underwent NCRT, and showed a good correlation between predicted survival probability and the actual survival rate. This model takes into account the clinicopathological factors, information about surgery, and postoperative morbidities. According to the total scores from the nomogram, the 5-year OS for patients could be divided into four groups at 57.3 %, 40.7 %, 18.3 %, and 6.1%, respectively, which differed from each other significantly. This model could also be used to evaluate specific survival rates. For example, a 49-year-old (0 points), male patient (65 points), with a tumor length of 5.5 cm (66 points), who reached a partial response (43 points), was negative for pathological lymph nodes, and had a tumor of 6 cm proximal margin length (0 points) with no anastomotic leakage (0 points) scores 173 points and yielded an estimated 5-year OS of 52 %. In this study, the nomogram showed good efficacy to predict prognosis and stratify risk. As mentioned above, although it had a similar stratification ability for 5-years OS with the AJCC guidelines, the nomogram performed better overall compared with the AJCC and RPA methods.

In this analysis, we use the criterion recommended in the NCCN guidelines to assess the response of the primary tumor to previous radiotherapy alone or chemoradiotherapy [26]. It turns out that primary tumor response is a strong influencing factor for survival. Donohoe et al. [27] reclassified the Mandard tumor regression grade (TRG) into three groups: TRG1 was equivalent to a complete response; TRG2/3 was equivalent to a partial response; and TRG4/5 was equal to a minimal response. Significant differences were revealed in terms of OS, DFS, and local recurrence among the three groups. By contrast, Dittrick et al. [28] found that pathological nonresponders after NCRT did not gain benefits in OS and DFS, or were even poorer compared with those that underwent surgery.

Tumor length, proximal margin length, and post-operative morbidities were also recognized as independent predictors in previous studies. Chao et al. [29] found that tumor length ≥ 6 cm was a predictor of local recurrence, while patients whose tumor length < 6 cm had a significant rate of recurrence after NCRT. Tumor length was also reported to be associated with effectiveness of chemo-radiotherapy (CRT ). Proximal margin length more than 5 cm *in vivo* was demonstrated to be an independent prognostic factor in R0 and R1 resection of esophageal cancer [30]. Preoperative morbidities, especially severe anastomotic leakage, could have an adverse effect on OS, DFS, and even locoregional recurrence [31]. A similar phenomenon was found in a meta-analysis of colorectal cancer [32]. Researchers hypothesized that an inflammatory response to anastomotic leakage might promote an environment that enhances cancer recurrence [33].

RPA models have been used in several other cancers, such as brain metastatic tumors, and are also a practical approach for stratification. However, in our study, the number of factors that could by used for stratification was limited and patients could only be divided into two subsets, which might have caused statistical bias. In comparison with the nomogram, the RPA model showed inferior stratifying accuracy and might need further modification before its application in esophageal cancer.

Our study had some limitations. First, data for the pretreatment clinical stage were not complete for some patients because of the lack of proper staging approaches in early years. Next, the time span for patients’ enrollment was more than three decades. Owing to advances in diagnostic and therapeutic techniques, patients enrolled in the 2000s had a better prognosis than those enrolled 1980s and 1990s (p < 0.05); however, the difference did not reach significance in multivariate analysis. In addition, this retrospective analysis attempted to predict survival using a nomogram, but requires further studies to validate and confirm the results.

In conclusion, this study established a prognostic nomogram model for TESCC patients who underwent neoadjuvant radiotherapy or chemoradiotherapy. The current AJCC staging system does not stratify prognostic groups adequately, nor does the RPA model. Age, sex, tumor length, tumor response, resection margin, proximal tumor length, lymph node status, and anastomotic leakage were identified as prognostic factors. Our study showed that the 5-year OS was 6.1–18.3 % for patients with a nomogram score of more than 270. More attention should be paid patients who are positive for positive lymph nodes after NCRT. A retrospective study showed that adjuvant chemotherapy might improve the prognosis of positive lymph nodes patients after NCRT, with an estimated 5-year OS of 41 % in the adjuvant group and 25% in the no adjuvant group (p = 0.033) [34]. This indicated that selected high risk patients after NCRT and surgery might need further treatment (e.g. adjuvant chemotherapy) to improve their survival.

## MATERIALS AND METHODS

### Patients

We analyzed retrospectively patients with previously untreated thoracic esophageal squamous cell carcinoma (TESCC) at the Cancer Hospital, Chinese Academy of Medical Sciences, from 1980 to 2014. Patients were staged as II–IV A according to the 6^th^ AJCC staging system. We adopt 6^th^ AJCC staging system in preoperative settings, and the 7^th^ AJCC system in postoperative settings because of the difficulty in determining the lymph node numbers by CT images. Those who were identified as having distant metastases were excluded. All the patients underwent neoadjuvant radiotherapy or chemoradiotherapy, followed by surgery. Baseline data, including demographic characteristics and clinicopathological characteristics, were obtained from medical records. The study protocol was performed in accordance with the guidelines outlined in the Declaration of Helsinki and was approved by the Ethics Committee of the Cancer Hospital, Chinese Academy of Medical Sciences. All patients provided written informed consent.

### Treatment modalities

Radiotherapy was delivered by conventional radiotherapy, 3-dimensional conformal radiotherapy, or intensity-modulated radiotherapy, at a median dose of 40 Gy (range, 28–70 Gy) and at 1.8–2.0 Gy per fraction, except for thirteen patients who received a simultaneously integrated boost to a median dose of 49.2 Gy (range, 40–60 Gy). Concomitant chemotherapy based on platinum and paclitaxel or 5-Fluoro Uracil was delivered either weekly or every 21 days. Surgery was carried out after a median of 4 weeks. Two-field lymph node dissection was performed routinely except for suspected or biopsy proven metastases in the supraclavicular lymph nodes; for such cases, three-field lymph node dissection was conducted. The treatment response of the primary tumor was classified according to the recommendation in the National Comprehensive Cancer Network (NCCN) guidelines. A complete response of a primary tumor was defined as < 1 % residual cancer cells; partial response was defined as 1–50 % residual cancer, rare individual cancer cells, or minute clusters of cancer cells; and minimal response was defined as more than 50 % residual cancer cells.

### Statistical analysis

The primary end point for the analysis was overall survival (OS), which was defined as the time from initial therapy of neoadjuvant radiotherapy or chemoradiotherapy to the following events: last follow-up or death. All the analyses were performed in the following three steps. First, Cox proportional hazards models were used to estimate the association between treatment and OS, with and without additional adjustment for potential confounders. Hazard ratios (HRs) and 95 % confidence intervals (CIs) were also calculated using the Cox proportional hazard model. The proportional hazard assumption was assessed using Cox models that allowed time-dependent HRs combined with a curve of log [--log(t)]. A nomogram and RPA model were then developed to classify patients after initial therapy with neoadjuvant radiotherapy or chemoradiotherapy into subgroups, given the selected prognostics factors. Finally, concordance probability and calibration were used to assess the performance of the models. We also used the bootstrap validation method to estimate the bias-corrected or overfitting-corrected predictive accuracy of the models, which was presented as a time-dependent receiver operating characteristic (ROC) curve and the concordance index (C-index). Calibration curves, which plotted the average Kaplan-Meier estimates against the corresponding nomogram for 5-year OS, were also provided to evaluate the discriminatory ability of the predictive models. The bootstrap-corrected 5-year OS was calculated by averaging the Kaplan-Meier estimates based on 200 bootstrap samples. All P values were two-sided, and P values less than 0.05 were considered statistically significant. All analyses were conducted in SPSS 20.0 for windows (SPSS, Chicago, IL, USA) or R 3.1.2(http://www.r-project.org/).

## Abbreviations

AJCC: American Joint Committee on Cancer
AUC: area under the curve
CI: confidence intervals
C-index: concordance index
DFS: disease free survival
HR: hazard ratios
NCCN: National Comprehensive Cancer Network
NCRT: neoadjuvant chemoradiotherapy
OS: overall survival
pCR: pathological complete response
ROC: receiver operating characteristic
RPA: recursive partitioning analysis
TESCC: thoracic esophageal squamous cell carcinoma
TRG: tumor regression grade
